# Letter from Dr. H. R. Hopkins

**Published:** 1884-07

**Authors:** H. R. Hopkins

**Affiliations:** Buffalo


### Correspondence.

*Editors Buffalo Medical and Surgical Journal:*

BUFFALO, JUNE 20, 1884.

Permit me to call attention to a fallacy in the position of the admirers of the term *functional diseases,* as shown in your recent editorial on that subject. It is proper to remark that the discussion to which you refer raised a two-fold objection to the use of this and similar medical terms of no certain sound, in that such terms were faulty in conception and pernicious in use. The evening in question furnished an illustration of such pernicious use.

The paper of the evening, presented by an eminent neurologist and supported by other equally prominent psychiatrists, referred to epilepsy as a chronic functional disease, and proceeded to point out its baleful effects upon reason and life, and in the way of treatment referred to the use of the trephine, of section of nerves, and ligation of the carotids and vertebrals.

In the discussion, it was urged that the term was a poor one for general or frequent use, and that when applied to epilepsy was particularly inappropriate.

The arguments of the minority are probably as well put as may be in your editorial, but would seem to be liable to the following criticism :

There is in your position no apparent recognition of the march of belief concerning the nature of disease from the times of the fathers, when nearly every disease was regarded as functional, until now, when the existence of such disease is generally denied.

We will, perhaps, avoid confusion by defining the term functional disease, and the remark may well be made that more exactness of definition would probably prevent discussion as to the propriety of its use.

It may reasonably be contended, that if retained at all, the term should be applied to disease having no morbid anatomy, that this is its natural meaning, and it was so used in early times.

To use the term as applied to disorders having no morbid anatomy, detectable by our present means of research, is to abandon the thought for which it originally stood and to put upon it a construction which makes the term a clumsy misnomer. You would then practically say, this is a functional disease, which may be a structural disease, but because we have not discovered its seat or the nature of its structural changes, therefore we say of it, that it is functional, although in so doing we disregard everything which we know of cause and effect and assume that here is an instance of effect without corresponding cause. We will, therefore, conclude that you are anxious to uphold the use of the term "functional disease," as the most appropriate designation of a class of disorders distinguished from others by the fact that they have no antecedent structural changes.

The gist of your argument and the quotations with which you would support it, seems to be that vital functions are exhibited by undifferentiated, structureless protoplasm, and that in a particular case a special instance or kind of protoplasm may exhibit a function so different from the normal in time, mode or degree as to constitute or cause disease, and that inasmuch as the protoplasm has no structure, it therefore can have no structural change, and the disease in question must be one of function.

You will permit me to remark, in passing, that the above is nearly all there is of the argument in favor of the soundness of the term "functional disease," and is a most glaring and vicious instance of reasoning in a circle.

To be more explicit. Upon page 520 you quote Foster's Physiology to establish the facts that the amœba is "almost wholly composed of undifferentiated protoplasm," and that this protoplasm "possesses certain fundamental, vital properties."

Upon page 522 you build upon the foundation thus laid after this fashion: "If an amœba composed of *structureless* protoplasm can set free the latent energy stored up in its substance, the difference in its susceptibility to external irritation must be one of function, for structural changes surely cannot occur where structure is absent." There is in this the following fallacy: You substitute structureless for undifferentiated.

In familiar conversation it is, perhaps, permissible to use these words as synonymous, but in the serious discussion of a scientific topic nothing could be more improper or faulty.

Herbert Spencer devotes page upon page to the work of acquainting us with the structure of undifferentiated protoplasm, and finds in this peculiar structure the power which shapes not alone the particular function of the part, but the processes of growth, repair and restoration as well. To be sure this structure is not a palpable fact, like the differentiation of the plant into stem, roots, leaves, flowers and seeds, or of an animal into nerves, muscles, blood and bones, but it, nevertheless, has as reliable a foundation as the molecular weights of the different elements.

Structureless, organic matter, capable of exhibiting the highest and most complex functions—capable of growth, of repair, and of reproduction, is at once the most ambitious and extravagant affirmation and negation, and easily outrivals the conception of the elephant supporting the earth from the back of the turtle. Such a proposition can scarcely be maintained by using words with a meaning altogether apart from that of the author, and although the precise relation of structure and function will doubtless ever remain an unsolved problem, yet every pertinent fact in biology plainly contributes to the support of the induction that complexity of function is the correlative of complexity of structure.

As to how slight a change a tissue must undergo before it can be said to suffer from structural change is probably a question not profitable to discuss, but in this connection it might be mentioned that the test of a normal tissue, or organ, is not applied by taking its weight, color, or microscopic appearance, but in all probability is more appropriately made by observing with what efficiency or otherwise it executes the function with which it is usually associated. As in chemistry matter is estimated by weight, so in physiology function is estimated by adaptation; and every fact in biology teaches us to believe that function is only exhibited by organized matter built up into more or less specialized structures.

Probably enough has been said to make it apparent that more pains must be taken to establish the existence of "structureless protoplasm" before proceeding to generalize upon its phenomena.

H. R. Hopkins.

